# Shifts in therapeutic practices and decline of medicinal cannabis in Indian North-Eastern Frontier (1826–1925)

**DOI:** 10.1186/s42238-022-00159-4

**Published:** 2022-09-28

**Authors:** Gita Bania

**Affiliations:** Department of History, University of Hyderabad, Hyderabad, India

**Keywords:** Cannabis, Medicinal cannabis, Hemp, India, Northeast Frontier

## Abstract

**Background:**

The emergence of colonial medicine in the North-Eastern Frontier witnessed different phases of consistent competition and resistance. Herbs such as cannabis provided native physicians with a coherent power to resist colonial medical intervention. Before British rule, cannabis assumed great significance in the socio-economic, cultural, and religious spheres. The colonizers’ bioprospection of cannabis shifted the production and use of cannabis from a medical and recreational plant to an industrial and commercial commodity. British policies on cannabis caused its ban leading to natives’ reliance on colonial cannabis products. As a result, the native medical practitioners resisted for reviving cannabis in the indigenous therapeutics. This paper mainly aims to investigate the decline of medicinal cannabis in indigenous therapeutics, causing subtle resistance of the native physicians of the North-Eastern Frontier.

**Methods:**

This paper follows a nomadology method based on primary and secondary sources to understand the impact on native physicians after the ban on private use, cultivation, and sale of cannabis. The primary sources/data have been collected from the Directorate of Archives: Government of Assam and Directorate of State Archives and Research Centre, Kolkata, West Bengal. Secondary sources have been collected from books, articles, and theses accessed from various libraries and websites.

**Results:**

Ban on cannabis led to dual responses from the indigenous population of the frontier. First is the interest of the native physicians resisting the revival of cannabis in indigenous therapeutics. The second is the interest of the frontier’s elites, who viewed cannabis as a “dangerous drug.” The British policies of control and restrictions on cannabis, the rift of response from the natives, and the over-powering of the indigenous therapeutics by the colonial medical system led to the decline of medicinal uses of cannabis in the North-Eastern Frontier.

**Discussions:**

Various pre-colonial and colonial factors helped colonial medical practices to get the upper hand over indigenous therapeutics. Such a shift led to the decline of indigenous medicinal cannabis causing native resistance, which was patient and silent.

**Conclusions:**

British ban on cannabis resulted in a rift of native responses, resistance, and decline of cannabis in the indigenous therapeutics of the North-Eastern Frontier.

## Introduction

The advent of the British East India Company to the Brahmaputra Valley materialized after signing the Treaty of Yandaboo in 1826, soon after the Second Anglo-Burmese War. Within a decade of annexing Assam, the Company spread its roots to engulf the entire North-Eastern Frontier. After annexation, the colonizers introduced various institutions such as knowledge, science, technology, and medicine to gain a strong hold over their colonized subjects (Arnold 1993; Goswami 2012). Colonial medicine in India developed in the nineteenth and twentieth century with the help of intensive explorations, investigation, research and study of topography, climate, and disease. It initially started to uphold the health of the European military. However, later on, colonial medicine spread to the native gentry, forming a means of direct intervention with the social, cultural, and material lives of the native population (Arnold 2000).

Colonial medical intervention in the North-Eastern Frontier was initially met with strict resistance and unacceptance. This was due to the natives’ reliance on indigenous therapeutics such as Ayurveda, Unani, and Folk medicine that used various herbal plants like cannabis (Chopra and Chopra 2009; Abrol 2006). As a result, various Acts were passed that curbed valuable plants like nux vomica, datura, opium, and cannabis by portraying them as “dangerous drugs” and forming a constant governmental check on the plants (Karmakar 2007; The Poisons Act, 1919-Indian Kanoon n.d.; Memorandum on Excise Administration in India so far as it is concerned with Hemp Drugs 1906; Mills and James 2003). This paper mainly deals with the British policies on medicinal cannabis and its subsequent impact on the native physicians, which caused contradictory responses and subtle resistance in the colonial North-Eastern Frontier.

After the Russian Hemp Blockade of 1807, the desire for hemp led to the influx of research undertaken by the East India Company’s Botanists, Surgeons, and Scientists (Davey 2012; Mills 2003). Cannabis was also provided to Messrs. Squire & Sons, Messrs. Burroughs & Wellcome Manufacturing Druggists, London, for medicinal and experimental purposes (Messrs. Squire, and Sons, London for Medical and Experimental Purposes 1902; Remission of-which it was intended to forward to Messrs. Burroughs & Wellcome Manufacturing Druggists 1897). Various scientists’ successful experiments and research identified Indian cannabis as the fiber-producing European hemp. These researches also revealed the significance of cannabis for medicines, fibers, and intoxicants, which assumed an important place in domestic and international markets (Transactions of the Agriculture and Horticulture Society of India 1839). The colonizers thereby took into their interest by dominating the cultivation, production, and trade of cannabis, connecting the remotest markets to the international ones.

Scientists Wood, Spivey, and Easterfield discovered one active ingredient of cannabis, which they named “cannabinol”in 1896 (Mills 2003). It was further confirmed by C.R Marshall, Professor of Medicine at the University of Cambridge, in 1903. He revealed that the substance could be further broken down, possessing no power to intoxicate. The discovery of the active ingredient of cannabis contributed to the cause of British Pharmacopeia. Cannabis was exported from British India to various Pharmaceutical Industries like Messrs. Squire & Sons, Messrs. Burroughs & Wellcome Manufacturing Druggists. By the late eighteenth and early nineteenth century, cannabis was included in Piso’s Cure Pharmaceuticals, One Day Cough Cure Pharmaceuticals, and Sears Roebuck Catalogues. Sajous’s Analytic Cyclopedia of Practical Medicine (1924) summarized cannabis’s use for treating migraines, gonorrhea, inflammation, tetanus, rheumatism, and many more ailments (Messrs. Squire, and Sons, London for Medical and Experimental Purposes 1902; Remission of-which it was intended to forward to Messrs. Burroughs & Wellcome Manufacturing Druggists 1897; Hand et al. 2016).

The three categories of cannabis research were viz. on its medicinal potentiality, fiber production, and the preservation and elevation of cannabis intoxicants. This was followed by various taxations, licenses, and druggist permits (Medicinal Properties of Ganja and Other Hemp Drugs 1872; Procedures Regarding the Preservation of Ganja 1920; Mills 2003). British India served as a supplier of raw materials exporting cannabis to various British and US Pharmaceutical Industries. British, once gaining control over the North-Eastern frontier easily traded cannabis to China, Tibet, and Burma (Bhuyan 1933). Cannabis trade with China was boosted after the 1911 Chinese Revolution, which banned the consumption of opium, resulting in a fillip in hemp drug (cannabis) consumption (Warf 2014). British ambitions on cannabis were achieved by making it a governmental possession. Such policies pushed back cannabis use in the indigenous therapeutics of the North-Eastern Frontier. Indigenous therapeutics, which showed signs of decay in contact with the emerging colonial medicine.

Prior to British rule, various works of literature, ancient texts, and medicinal compendium confirmed the therapeutic use of cannabis throughout India (Khanikar 2011; Karpakal 2017; Grierson 1894; Evidence Given Before the Committee Appointed to Enquire into Certain Aspects of Opium and Ganja Consumption 1913; Report of the Committee Appointed to Enquire into Certain Aspects of Opium and Ganja Consumption 1913). Cannabis has been used as a medicinal herb in Ayurveda to treat ailments like indigestion, pain, skin diseases, sexual debility, and chronic diarrhea. For the first time, it was mentioned in the Atharvaveda as “one of the five sacred plants on the planet Earth.” Sushruta Samhita also mentioned cannabis as a remedy for diarrhea, catarrh, and fever from phlegm and bile. The medicinal potentiality of cannabis is also evident from a reference in Vārttikā and Aṣṭādhyāyi of Pāṇini (Karpakal 2017; Grierson 1894). However, during the colonial period (1757–1947), we find several pieces of literature on cannabis partially depicting it as an “intoxicant drug” linked with “oriental degeneracy, madness and crime” (Mills 2003; Herer 2006). Oriental degeneracy in this context implies degeneracy of colonial Asian countries including British India. Cannabis was addressed as “Indian hemp” in many literatures. It denoted that the “degraded” practice of cannabis intoxication originated from India (Fankhauser 2008).

The first European to make a compilation of the Indian materia medica was D’Orta in his book “Coloquios dos simples e drogas he cousas medicinais da India”. This work described that bangue (cannabis) was consumed by the Indians for venereal acts, and used by the armies’ officers and soldiers for instant refreshment and sleep. Another work on cannabis in The Natural History of India by Christoval Acosta described the use of cannabis for enhancing appetite and treating insomnia. Robert James, the author of Medicinal History, also mentioned similar accounts of cannabis with the same stories of “soldiers, sex and sleep.” All these texts revealed a repeated trend of duplicate accounts, which, however, lacked proper knowledge of the medicinal use of cannabis before the 19th century (Mills 2003). Finally, a halt to this trend was brought by Ainslie’s work of Materia Medica of Hindoostan. He described medicinal cannabis for treating piles and diarrhea, and also as a painkiller. However, the most descriptive and significant account of cannabis was provided by W.B O’ Shaughnessy Professor of Materia Medica, Medical College of Calcutta, in his work On the Preparations of Indian Hemp or Gunjah (cannabis indica). His work describes the history of “Indian hemp,” its use, botanical characteristic, chemical and medicinal properties, and its production, including its various derivatives (O’Shaughnessy 1843). The report of Shaughnessy led to an influx of research and experiments conducted by several scientists, chemists, and pharmacologists on medicinal cannabis.

Moreover, the works of scientist W.Ley, Dr. Prain (Curator of Herbarium, Royal Botanic Garden, Sibpur), Mr. Jenks, Chemical Examiner for Customs and Excise, Bengal, requires special mention, which contributed further to the medicinal and intoxicant research on cannabis during the colonial period (Report of the Cultivation and Use of Ganja 1893; Ley 1843).

More recent works on cannabis have been aptly provided by Jack Herer, James H. Mills, Bradley J. Borougerdi, Manfred Fankhauser, Robin Room et al., Dana Zarhin, and Sharon R. Sznitman, John Collins, Kenzi Riboulet-Zemouli et al., and Matt Stolick (Borougerdi 2018; Room et al. 2010; Zarhin et al. 2020; Collins 2020; Zemouli et al. 2019; Stolick 2009). However, Mills’ works stand out to give a historical context of cannabis by placing it in the economic, political, and policies of the British Indian Empire (Mills 2003, 2000, 2004). Poonam Bala has provided another seminal work on the medical history of Bengal. Her work lays out the encounter between colonial medicine and the indigenous medical practices, which mainly led to the depreciation of the latter in due course of time (Bala 1991). The cause of reviving indigenous therapeutics was eventually taken up by the nationalists who viewed its link with the cultures of India’s past. However, the nationalist’s zeal for reviving indigenous medicine did not touch medicines like cannabis, which the colonizers portrayed as a “dangerous drug.” This raises an impediment question regarding what led to such discrimination and contradictory responses from the natives relating to cannabis and its subsequent decline in the indigenous therapeutics of the frontier?

### Cannabis and British policies

Britain’s initial search for hemp (a male derivative of cannabis) in India began after the Russian Hemp Blockade in 1807. Russia was bound by the Treaty of Tilsit, banning all Anglo-Russian trade. This led to the influx of researchers, surveyors, scientists, and botanists to Indian colonies searching for cannabis. Their explorations emphasized Indian cannabis as the fiber-producing European hemp (Transactions of the Agriculture and Horticulture Society of India 1839; Mills 2003; Davey 2012). The three categories of research were viz. cannabis fiber production, medicinal research, and the preservation and elevation of cannabis intoxicants. The colonial bioprospection of cannabis was successfully conducted by Robert Hooke, Ainslie, W.B O’ Shaughnessy, W.Ley, R. Rowan Lees, Jacques Joseph Moreau, Wood, Spivey, and Easterfield (O’Shaughnessy 1843; Ley 1843; Mills 2003; Logan 1974; Lees 1895). Bioprospection is the observations, explorations, and study of valuable tropical plants, and herbs mainly aimed for large scale plantation for greater commercial profits (Chakrabarti 2014, 34). The contributions of the researchers and scientists included cannabis in the British Pharmaceutical Codex 1934 (Small 2017; Waring 1885; British Pharmaceutical Codex 1934). Although such research gave a fillip to the British Pharmaceutical Industries, it pushed back the use of cannabis in the indigenous therapeutics of the frontier by placing cannabis under the strict governmental gaze. This was coupled with various taxations, the introduction of licenses and druggist permits, and the imposition of criminal charges on the offenders (Memorandum on Excise Administration in India so far as it is concerned with Hemp Drugs 1926; Evidence of Witnesses from Bengal and Assam taken before the Indian Hemp Drug Commission 1894). The sale of hemp drugs required special licenses in Assam, which were issued by the Naugaon Ganja Cultivators Cooperative Society Limited in Rajshahi. Licenses were obtained by auction for a period of one year. Apart from these, retail and wholesale licenses and three druggist permits were issued between 1912 and 1913 in Sylhet, Kamrup, and Sibsagar (in Assam, India) for medical preparations. Druggist permits exceeded in the subsequent years, from 1923 to 24 and 1924 to 25 from nine to seventeen (Memorandum on Excise Administration in India so far as it is concerned with Hemp Drugs 1926). However, before British control, medicinal cannabis was either locally grown or easily accessible in the daily and periodic “hats” (markets) in Assam and even in the yearly fairs, like Jonbeel Mela (Goswami 2012). Moreover, India served as a supplier of raw materials exporting cannabis to various British and US Pharmaceutical Industries like Messrs. Squire & Sons, Messrs. Burroughs & Wellcome Manufacturing Druggists, William Ransom & Sons Ltd., and Parke Davis & Co. (Hand et al. 2016, Messrs. Squire, and Sons, London for Medical and Experimental Purposes 1902; Remission of-which it was intended to forward to Messrs. Burroughs & Wellcome Manufacturing Druggists 1897).

Towards the end of the nineteenth century, a hitched issue arose regarding cannabis consumption causing insanity and crime. This was followed by the Indian Hemp Drug Commission survey, 1893–1894 (now IHDC) (Mills 2003; Marijuana and Health 1971). As the lunatic asylums were filled up by all ganja smokers, it compelled the Government to establish the Indian Hemp Drug Commission (IHDC) to investigate the issue (Shamir and Hacker 2001; Indian Hemp Drug Commission 1894; Final Report of the Royal Commission on Opium 1895; Mills 2006). The relation of cannabis consumption with crime was viewed to be caused by the loss of control under intoxication leading to unlawful acts. The IHDC stated that excessive intoxication of hemp caused insanity. In criminal cases, the natural causes were unknown (Report of the Indian Hemp Drug Commission 1893-94 1894). However, IHDC had its limitations. Out of fifty-seven medical practitioners, only six were Ayurvedic specialists, and most of the witnesses were Company’s officers or employers, which limited its reliability (Basu 2000). IHDC also ignored the socio-cultural and religious significance of cannabis throughout India. Instead, it mainly focused on “cannabis, madness, and crime.”

As the British, towards the end of the nineteenth century, successfully carried on a flourishing trade of exporting opium and hemp drugs to China, Tibet, Burma, Egypt, the US, and South African countries, it became a matter of great condemnation in the international circles, resulting in the League of Nation’s Advisory Council on Opium. The “League of Nations” was followed by two opium conferences of 1924–1925.

Although the main focus was on opium, cannabis exploded into the agenda in the Second Opium Conference under Egyptian pressure as imposed by EL Guindy (Egyptian delegate), backed by the US and China. These countries demanded the trade of opium and hemp drugs to be outlawed. Britain thereby agreed to halt the hemp trade to the dissented nations unless on the provision of a certificate for industrial and medicinal use. However, Britain did not ban hemp drugs but made it a complete governmental possession. As a result, there was a considerable decline in the indigenous therapeutic use of cannabis and its derivatives (Mills and James 2003).

### Native responses toward ban of cannabis

The indigenous medical practitioners despised the dominance of colonial medicine. In this connection, they provided subtle resistance to the colonial medical intervention by trying to revive declining herbs like cannabis in the indigenous therapeutics of the frontier. In this connection, several vernacular works on medicine were published (Gait 2010; Barooah 1978; Das 2010; Khanikar 2011; Katoki 2009; Sonovāl 2013).

As the indigenous therapeutics were viewed to be linked with the “cultures of the past,” the cause of revival of the indigenous therapeutics was taken up by the nationalists. Moreover, during the Swadeshi Movement in 1905, the agenda of using national goods and boycotting foreign products took shape in the frontier. As a result, the consumption of ganja reduced considerably (Trivedi 1995; Assam Congress Opium Enquiry Committee 1925). The Swadeshi Movement and the Non-Cooperation Movement led to the decline of ganja consumption. It decreased to 0.26 tolas (3.032 g) in the Brahmaputra Valley in 1905–1906, and during the Non-Cooperation Movement, it further dropped down from 632 maunds 29 seers (23,588.894 kg) in 1920 to 452 maunds 18 seers (16,870.53 kg) in 1921–1922 and 1923–1924, the consumption further decreased to 344 maunds (12839.52 kg), which were 45.7% less than in 1920–1921 (Assam Congress Opium Enquiry Committee 1925; Report of the Indian Excise Committee (1905-06) 1907; Deshpande 2009).

Cannabis was looked down upon by the “elites.” Gandhi’s idea of village Swaraj was that “if more land is available, it will grow useful money crops excluding ganja, opium and the like” (Prabhu and Rao 1967). Such an idea of the partial portrayal of cannabis got embedded in the minds of the frontier elites. This led to inconsistent frictions between the sectional and the general interests. Sectional interests were represented by the elites standing against cannabis, and general interests were represented by the natives of the frontier raising subtle resistances against the control and restriction of cannabis. Moreover, IHDC revealed reports of cannabis being used as a weapon of caste discrimination. The Hindu caste system has four custer viz. *Brahmin*, *Kshatriya*, *Vaishya*, and *Shudra*. However, at the village and local level, these four castes are further divided into smaller sub-castes known as *jati* (Khatoon 1995). The consumption of cannabis was linked with the lower castes (*Sudras* and outcastes), and the upper castes (*Brahmin*) claimed to vehemently despise cannabis use. A member of the Indian Hemp Drug Commission stated the following:

There can be no other agency in bringing to light the history of a lunatic than the police, because ganja-smokers are generally men of low-caste and of bad character, with whom the high officers cannot be in touch. The habit of ganja smoking is looked down, and therefore those who use ganja smoke it secretly, trying their best to conceal the fact from their elders and their society. (Report of the Indian Hemp Drug Commission 1893-94 1894, 413).

Such a rift in response from the natives of the frontier was due to the elites’ inclination towards westernization, which infused a negative impression on cannabis. However, prior to British rule, cannabis was used by all castes (Shivaharidas 2012). This evidently, ignored the unabated struggles and resistances of the natives under the British rule.

## Conclusion

Such a shift and degeneration of indigenous medicinal cannabis was due to the decline of indigenous therapeutic practices, primarily due to the lack of initiatives undertaken by the British Government. Although both the indigenous and the modern therapeutics were patronized differently, British preferred the development of colonial medicine, providing a blow to the indigenous medical practices. Moreover, at the initial stage, British followed a policy of recruiting both Indian and European scholars to amass the indigenous medicinal knowledge, which later contributed to the cause of British Pharmacopeia. They also did not provide any registration or recommendation to any indigenous medical practices. These policies of amassed knowledge boosted the colonizers’ intervention, which mainly resulted in their monopoly over Indian drugs and trade (Arnold 2000). Secondly, the decline of indigenous therapeutics could not be solely imposed on the colonizers. Degeneration of the indigenous medical practices could be traced back to the ancient period, when medicine was claimed to be practiced by the lower caste of the society. However, due to the rigidity of the caste system, the *Brahmanas* were viewed as the bearers of “knowledge” with their grip over therapeutic practices. They did not allow any relevant enquiry or systematic development in the indigenous therapeutics. This is reflected in their codification of Ayurveda revealing, the dominance of religion and superstitions (Bala 1991). Thirdly, there was no uniform medical system due to various foreign interventions. During the medieval period, India came to be dominated by the Unani system of medicine. The Muslim royalty only recognized *hakeems* brought from Persia, viewing the conservative nature of ancient medicine. *Hakeem* is a Unani physician. Unani is a system of medicine derived from medieval Muslim physicians from Byzantine Greece. Similarly, in the Brahmaputra Valley, the Ahom royalty’s trust for the Muslim healers and Ayurvedic *vaidyas* (Ayurvedic medical practitioner) was predominantly less as they gave more importance to Folk-medicine. Thus, these changes, interventions, and the dominance of external factors over indigenous therapeutics ultimately led to the over-powering of the pre-modern by the modern medical practices under British rule.

Moreover, the contradictory response of the natives regarding the ban on cannabis was mainly due to the elites’ acceptance of British “modernity.” The elites tried to form their brand of “Indian modernity” through the selective incorporation in indigenous knowledge and traditions. Their prime objective regarding the native medical practices was to provide a more scientific base detached from the dominance of religion and superstitions. However, by denouncing cannabis, they demonstrated “selective appropriation” in the indigenous medical systems.

The general use, cultivation, and “smuggling” of cannabis were also evident in British colonies of America, China, Africa, Sri Lanka, and Burma. By global standards, cannabis regulations in Africa were early and rigorous, targeting specific socio-economic and cultural groups (Hand et al. 2016). In Sri Lanka, the Opium and Bhang Ordinance, enacted by the British authority in 1867, limited the selling of cannabis to licensed sellers only. The import of bhang, also known as ganja, was prohibited in 1897. The British government introduced the Indian Hemp Ordinance in 1905. In 1907, producing, importing, or distributing cannabis was punishable by a 100-rupee fine and 6 months in prison. Despite these restrictions and control on cannabis, people continued its use. We can also find many medicinal books published during the same period. In Sri Lanka, the medicinal significance of cannabis has been included in various books (Weliange 2018).

Many nations have already taken the required actions to solve the war on cannabis, but India is still in a time-warp. This calls for understanding the relevance of government regulations on cannabis and the general masses’ interests to break off this ageless bigotry.

## Data Availability

The author used both primary and secondary sources in this manuscript. The primary sources/data has been collected from the Directorate of Archives: Government of Assam; Directorate of State Archives and Research Centre, Kolkata, West Bengal; National Library of India; Guwahati District Library, and Nabin Chandra Bordoloi Library. Secondary sources have been collected from books, articles, and reports accessed largely from Guwahati District Library, Krishna Kanta Handique Library, Guwahati, Lakshminath Bezbaroa Library, Indira Gandhi Memorial Library, University of Hyderabad, and the various referred websites viz.: http://ia801609.us.archive.org/1/items/in.ernet.dli.2015.210881/2015.210881.The-British.pdf 10.1007/978-3-642-22881-0 http://www.sih.sagepub.com/content/25/1/109 10.2307/2213324 http://www.linkedin.com/pulse/cannabis-one-sacred-plant-atharva-veda-sindhu-karpakal https://indiankanoon.org/doc/1399304/ http://www.jstor.org/stable/25491786 https://www.emcdda.europa.eu/publications/monographs/cannabis-volume1_en http://www.jstor.org/stable/829081

## Abbreviations

CO.: Company
E.B: Eastern Bengal
ETC: Et cetera
IHDC: Indian Hemp Drug Commissions
Ltd.: Limited Company
MESSR: Messieurs is used before the names of two or more men as part of a business
UK: United Kingdom
US: United States
Vol.: Volume
Viz: Videlicet (Latin) which is a synonym for “namely”
